# Appeals

**Published:** 1919-01-25

**Authors:** 


### Appeals.

### Matinee for Seamen's Hospital.

ON Friday this week Mr. C. Gulliver is giving a matinée at the London Palladium in aid of the Seamen's Hospital. Greenwich. The programme will be contributed to by about a dozen of the best known vaudeville performers of the day. The King has sent a cheque to Mr. E. Gray, hon. organiser of the matinée, with instructions to invite a number of sailors to the performance. Others are invited to follow His Majesty's example. All are interested in the sailor; here is a chance to show practical sympathy.

### Charing Cross Hospital Dance.

LADY (MILSOM) REES has arranged a dance for February 5, in the Grafton Galleries, in aid of the funds of the Charing Cross Hospital. Tickets, one guinea each, may be obtained from the Secretary of the hospital. This is the hospital's centenary. For upkeep alone money is needed; there are no ambitious building schemes.

